# Effects of *APOE* ε2 on different cognitive domains in the PUMCH dementia cohort

**DOI:** 10.1002/alz.087946

**Published:** 2025-01-03

**Authors:** Li Shang, Liling Dong, Chenhui Mao, Xinying Huang, Shanshan Chu, Wei Jin, Tianyi Wang, Bo LI, Jialu Bao, Yuyue Qiu, Yuhan Jiang, Yixuan Huang, Jing Gao

**Affiliations:** ^1^ Peking Union Medical College Hospital, Beijing China; ^2^ Peking Union Medical College Hosital, Beijing, Bei Jing China

## Abstract

**Background:**

Previous studies on *APOE* have mostly focused on *APOE* ε4, while less attention has been paid to *APOE* ε2. The aim of this study was to clarify the effect of *APOE* ε2 on different cognitive domains in dementia patients.

**Method:**

All subjects were from the Peking Union Medical College Hospital (PUMCH) dementia cohort and included clinical diagnoses of AD, VaD, FTLD, and LBD. All included individuals had completed the neuropsychological assessment. Genotypes were used to bin participants into five groups based on carrier status: *ε2* carriers (*ε2/ε2* or *ε2/ε3*), *ε2/ε4* carriers, *ε3/ε3* carriers, *ε3/ε4* carriers, and *ε4/ε4* carriers. The effect of *APOE* ε2 on different cognitive domains of the patients with dementia was assessed by using multivariate regression models.

**Result:**

A total of 348 dementia patients were included in this study, with a mean age of 68.4± 9.8 years, 69.3% with AD, 14.4% with VaD, 10.1% with FTLD, and 6.3% with DLB. 19.5% were *ε2* carriers, 3.4% were *ε2/ε4* carriers, 30.7% were *ε3/ε3* carriers, 32.3% were *ε3/ε4* carriers, and 14.1% were *ε4/ε4* carriers. Compared to *ε3/ε3* carriers, *ε2* carriers were more successful in MOCA (β = 1.93, P = 0.016), trail making test part A (β = 1.18, P = 0.025), nonverbal memory (β = 1.08, P = 0.003), AVLT 5 (β = 0.27, P = 0.039), episodic memory (β = 0.41, P = 0.005), calculation (β = 1.05, P = 0.045) and word fluency (β = 1.82, P = 0.03). Compared to *ε4/ε4* carriers, *ε2* carriers performed better in nonverbal memory (β = 1.59, P<0.001), AVLT 4 (β = 0.68, P<0.001), AVLT 5 (β = 0.62, P<0.001), associative learning test (β = 0.57, P<0.001), episodic memory (β = 0.46, P = 0.007) performed better. When adjusted for clinical diagnosis, the above significant differences remained statistically significant. While *ε2/ε4* carriers performed better in associative learning (β = 1.38, P<0.001) compared to *ε3/ε3*, visuospatial (β = ‐1.94, P = 0.020) function was worse than *ε3/ε3* carriers. Compared to *ε4/ε4*, *ε2/ε4* carriers performed worse in similarity test and visuospatial function, while in verbal memory performed better

**Conclusion:**

The present study found complex effects of *APOE ε2* and *APOE ε2/ε4* on cognitive domains that varied according to the selected control group, whereas different clinical diagnoses did not seem to influence their effects.

